# Comparison of ^68^Ga-DOTANOC and ^18^F-FDOPA PET/CT for Detection of Recurrent or Metastatic Paragangliomas

**DOI:** 10.1148/rycan.240059

**Published:** 2024-12-06

**Authors:** Linjie Bian, Junyan Xu, Panli Li, Liyan Bai, Shaoli Song

**Affiliations:** From the Department of Nuclear Medicine, Fudan University Shanghai Cancer Center, 270 Dongan Road, Xuhui District, 200032 Shanghai, China; and Shanghai Engineering Research Center of Molecular Imaging Probes, Shanghai, China.

**Keywords:** ^68^Ga-DOTANOC, ^18^F-FDOPA, Pheochromocytoma, Paraganglioma

## Abstract

**Purpose:**

To evaluate the diagnostic performance of gallium 68 (^68^Ga)-DOTA-NaI3-octreotide (^68^Ga-DOTANOC) and fluorine 18 (^18^F)-fluoro-l-3,4-dihydroxyphenylalanine (^18^F-FDOPA) PET/CT in detecting recurrent or metastatic paragangliomas.

**Materials and Methods:**

This single-center retrospective study included patients with paragangliomas who underwent both ^68^Ga-DOTANOC PET/CT and ^18^F-FDOPA PET/CT between August 2021 and December 2023. The diagnostic performance of these two tracers in detecting recurrent or metastatic tumors was compared using several metrics, including sensitivity, negative predictive value, and accuracy.

**Results:**

This study included 36 patients (median age, 52 years [range, 14–78 years]; 16 female, 20 male). Of these, nine underwent initial ^68^Ga-DOTANOC and ^18^F-FDOPA PET/CT examinations before treatment, and the remaining 27 underwent posttreatment examinations. Twenty-two of those 27 patients had recurrence or metastasis. According to lesion-level analysis, ^68^Ga-DOTANOC had higher sensitivity, negative predictive value, and accuracy for diagnosis of bone metastases than did ^18^F-FDOPA PET/CT (97% vs 78% [*P* < .001], 85% vs 42% [*P* = .02], and 97% vs 81% [*P* < .001], respectively). ^18^F-FDOPA PET/CT had higher sensitivity, negative predictive value, and accuracy for the diagnosis of liver metastases than did ^68^Ga-DOTANOC PET/CT (73% vs 15% [*P* < .001], 68% vs 41% [*P* = .04], and 83% vs 46% [*P* < .001], respectively). According to patient-level analysis, the sensitivity of ^18^F-FDOPA PET/CT for diagnosing liver metastases was higher than that of ^68^Ga-DOTANOC PET/CT (88% vs 25%; *P* = .04).

**Conclusion:**

In patients with recurrent or metastatic paragangliomas, ^68^Ga-DOTANOC PET/CT showed better performance than ^18^F-FDOPA PET/CT in detecting bone metastases, and ^18^F-FDOPA PET/CT performed better in detecting liver metastases.

**Keywords:**^ 68^Ga-DOTANOC, ^18^F-FDOPA, Pheochromocytoma, Paraganglioma

Published under a CC BY 4.0 license.

See also commentary by Burkett and Johnson in this issue.

## Graphical abstract

**Figure:**
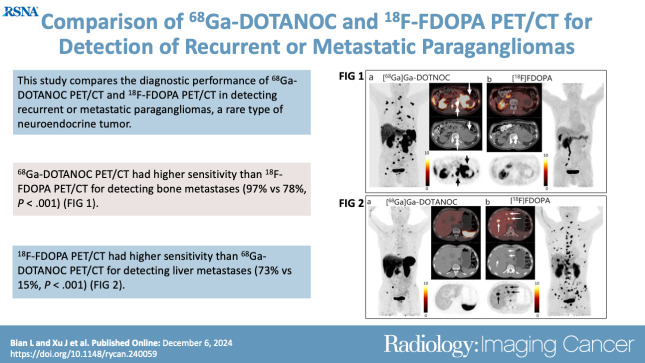

**Summary**

In patients with recurrent or metastatic paragangliomas, ^68^Ga-DOTA-NaI3-octreotide PET/CT showed higher sensitivity than ^18^F-fluoro-l-3,4-dihydroxyphenylalanine (^18^F-FDOPA) PET/CT for detecting bone metastases, and ^18^F-FDOPA PET/CT was more sensitive for detecting liver metastases.

**Key Points**

■ For the detection of bone metastases in patients with recurrent or metastatic paragangliomas, ^68^Ga-DOTA-NaI3-octreotide (^68^Ga-DOTANOC) PET/CT had higher sensitivity than ^18^F-fluoro-l-3,4-dihydroxyphenylalanine (^18^F-FDOPA) PET/CT (97% vs 78%; *P* < .001).
■ ^18^F-FDOPA PET/CT had higher sensitivity than ^68^Ga-DOTANOC PET/CT for detecting liver metastases (73% vs 15%; *P* < .001).

## Introduction

Paragangliomas (PGLs) are rare neuroendocrine tumors that originate from neural crest tissue and can cause refractory hypertension. PGLs located in the adrenal medulla are referred to as pheochromocytomas (PCCs) (1,2). PCCs and paragangliomas (PPGLs) are characterized by recurrence, metastasis, and tumor heterogeneity (3). Benign and malignant tumors cannot be differentiated on the basis of histopathologic findings (4); therefore, imaging examinations are particularly important for precise treatment. Current imaging methods include anatomic imaging, such as CT and MRI, and functional imaging that relies on physiologic processes or receptor targeting. However, metastatic lesions and local recurrence may be difficult to detect with CT and MRI, and functional imaging usually has higher sensitivity (5,6).

The gallium 68 (^68^Ga)–labeled somatostatin analogue (SSA) is a 14–amino acid peptide hormone that binds to the somatostatin receptor (SSTR) (7). Because of the ability of PPGLs to express high-level SSTR, they can be specifically recognized by radioactive-labeled SSAs (8). Fluorine 18 (^18^F)-l-3,4-dihydroxyphenylalanine (^18^F-DOPA) is an analogue of levodopa and a precursor of dopamine neurotransmitters. Catecholamine synthesis is active in neuroendocrine tumors, leading to tumor uptake of ^18^F-FDOPA (9). ^18^F-FDOPA PET can thus be used for the diagnosis of neuroendocrine tumors, such as PPGLs (10). ^68^Ga-DOTA-NaI3-octreotide (^68^Ga-DOTANOC) targets tumors with high SSTR expression, whereas ^18^F-FDOPA reflects catecholamine metabolism. Thus, these tracers may differ in detecting recurrent or metastatic paragangliomas.

Because of the relative rarity of PPGLs, few studies have evaluated the use of both ^68^Ga-DOTANOC and ^18^F-FDOPA tracers in PPGLs. Even fewer studies have focused on metastatic lesions and postoperative recurrent lesions. Evaluation of metastases and postoperative recurrence is crucial because these lesions often arise in anatomic locations with significant physiologic uptake, such as the liver, making it more challenging to distinguish them from normal tissues. In addition, recurrent lesions may be located in regions with postsurgical changes or fibrosis, further complicating detection. Therefore, it is essential to assess how different tracers, such as ^68^Ga-DOTANOC and ^18^F-FDOPA, perform in these complex scenarios to improve diagnostic accuracy. The purpose of this study is to compare the diagnostic performance of ^68^Ga-DOTANOC and ^18^F-FDOPA for detection of recurrent or metastatic PPGLs.

## Materials and Methods

### Patient Selection

This single-center retrospective study was approved by the committee of Fudan University Shanghai Cancer Center (ethical approval number: 1612167–18). All patients provided written informed consent and agreed to participate in this study.

The study included patients with PPGL who were admitted to our hospital from August 2021 to December 2023. The inclusion criteria were as follows: *(a)* histologically confirmed diagnosis of PPGLs requiring further restaging; *(b)* suspected metastasis shown at conventional imaging (CT, MRI, US); *(c)* two examinations with ^18^F-FDOPA PET/CT and ^68^Ga-DOTANOC PET/CT, with imaging intervals of less than 2 weeks; and *(d)* complete medical imaging (CT or MRI), clinical information (eg, physical examination, treatment modalities), and laboratory tests (eg, tumor markers) during follow-up. The interval between examinations of less than 2 weeks was selected on the basis of a previous study (11) and ensured that comparisons between each examination type were not compromised by disease progression. Patients with other malignant tumors were excluded.

### Imaging Protocol

^68^Ga was eluted from a ^68^Ge generator (IGG100; Eckert & Ziegler), and ^18^F was obtained from a Siemens RDS Eclipse Medical Cyclotron. The radiochemical purity of ^68^Ga-DOTANOC and ^18^F-FDOPA was greater than 95%.

For ^68^Ga-DOTANOC PET/CT, no fasting or blood glucose control was required. Before drug injection, 20 mg of furosemide and 2 MBq/kg of ^68^Ga-DOTANOC were injected intravenously. Sixty minutes after injection, whole-body PET/CT was performed.

For ^18^F-FDOPA PET/CT, entacapone (0.2 g) was administered 1 hour before intravenous injection of ^18^F-FDOPA (3.5 MBq/kg) to reduce peripheral nervous system uptake of ^18^F-FDOPA; PET/CT was performed 90 minutes later.

Briefly, low-dose CT was first performed using the Biograph mCT Flow PET/CT system, with a tube voltage of 120 kV and a current of 140 mA. The scanning layer thickness and spacing were both 3 mm. Subsequently, PET was performed, with a collection time of 3 minutes for each bed. CT images were used to iteratively reconstruct PET images for attenuation correction; after acquisition, the ordered subset expectation maximization method was used for image reconstruction.

### Imaging Interpretation

^68^Ga-DOTANOC and ^18^F-FDOPA PET/CT images were analyzed independently by two experienced nuclear medicine physicians (J.X. and P.L., with 13 years and 8 years of experience), who were blinded to the other PET scans and other clinical information (including CT, MRI, endoscopy, and pathologic results). Disagreements were resolved by consensus between the two readers. Metastatic lesions were defined as the presence of metastases in nonchromophilic tissues, such as lymph nodes, bone, liver, and lungs. During visual analysis, a positive lesion was defined as a focal uptake level exceeding the corresponding background level, excluding physiologic or known benign lesions with high SSTR expression or DOPA hypermetabolism. Lesions were considered malignant during follow-up on the basis of *(a)* typical malignant features (ie, mass, abnormal density, poor circumscription, and destruction) and *(b)* a substantial reduction or progression in size after anticancer treatment confirmed with follow-up imaging (ie, CT and MRI) according to Response Evaluation Criteria in Solid Tumors 1.1 (11). The maximum standardized uptake value (SUV_max_) for each patient was calculated by placing a spheroid-shaped volume of interest within the lesion. The mean SUV of normal tissue was recorded as background uptake. The tumor-to-background ratio (TBR) was calculated according to the following formula: TBR = SUV_max_ of lesion/SUV_mean_ of background. The location and quantity of lesions were recorded. If the number of lesions in a region (region refers to lung, bone, liver, and lymph node metastases) exceeded five, the count was truncated to five to avoid bias (11).

According to the World Health Organization classification criteria, PGLs were divided into three categories based on location: head and neck paraganglioma (HNPGL), PCC, and extra-adrenal PGL in the chest and abdomen (2).

### Reference Standard

Positive lesions were confirmed with pathologic examination. All pathologic examinations were conducted by experts with more than 10 years of experience in the neuroendocrine field. For lesions without pathologic findings, a composite reference standard was used, which was synthesized by combining anatomic and functional imaging and follow-up (≥3 months).

### Statistical Analysis

Statistical analyses were performed using SPSS software, version 25.0 (IBM). Continuous data are presented as means ± SDs or medians and ranges, and categorical data are presented as numbers and percentages. The sensitivity of ^68^Ga-DOTANOC and ^18^F-FDOPA PET/CT for the diagnosis of paragangliomas at different sites was compared using the McNemar test. Sensitivity, specificity, positive predictive value, negative predictive value, and accuracy were compared using the McNemar test to assess the diagnostic performance in detecting primary tumors and local recurrence, lymph node metastasis, bone metastasis, liver metastasis, and lung metastasis. For comparisons of ^68^Ga-DOTANOC and ^18^F-FDOPA PET/CT in patients with *SDHx*-related and non–*SDHx*-related PPGLs, the McNemar test was also applied to evaluate differences in diagnostic sensitivity. Paired *t* tests were used to compare clinical variables between the two groups. *P* values less than .05 (two-sided) were considered to indicate statistically significant differences.

## Results

### Patient Characteristics

**Figure 1:**
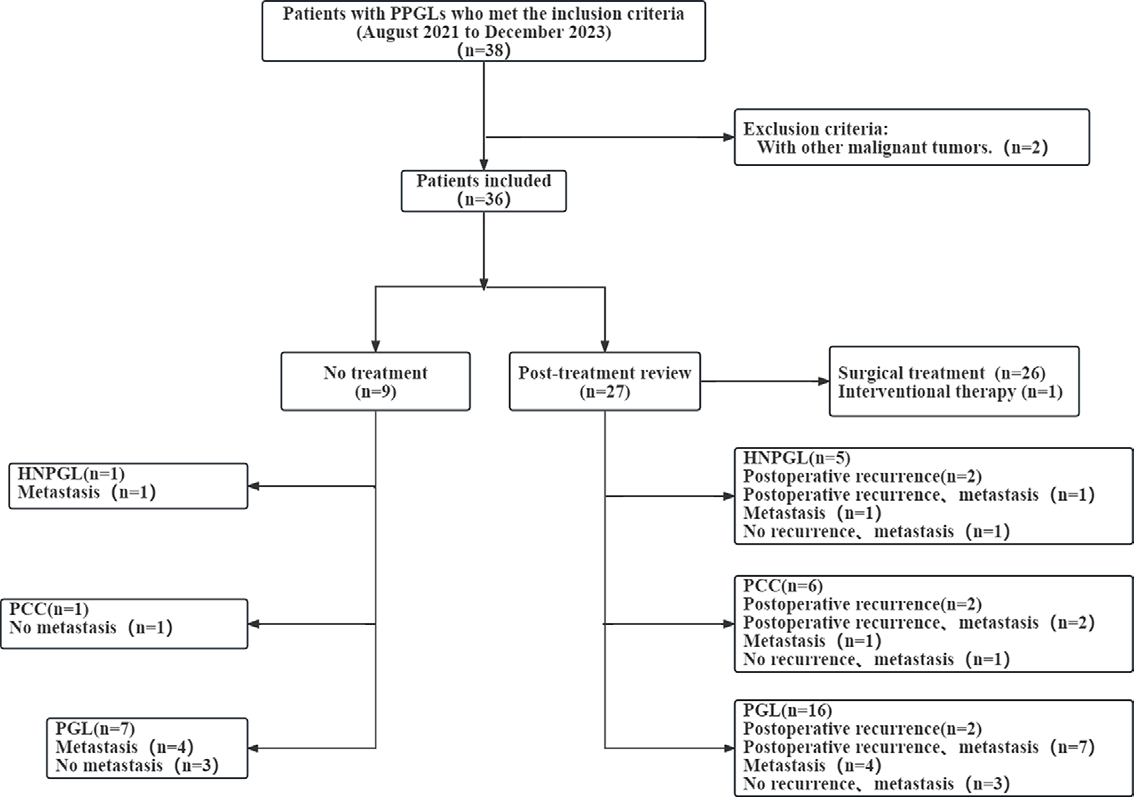
Flow diagram of patient selection for this study. HNPGL = head and neck paraganglioma, PCC = pheochromocytoma, PGL = paraganglioma, PPGL = pheochromocytoma and paraganglioma.

This study included a total of 36 patients (16 female and 20 male; median age, 52 years [range, 14–78 years]). Of these, nine patients underwent their initial ^68^Ga-DOTANOC PET/CT and ^18^F-FDOPA PET/CT examination before treatment, and the remaining 27 patients underwent posttreatment examinations. Among the 27 posttreatment patients, 22 had recurrence or metastasis (median time to progression, 25 months [range, 1–216 months]), and five patients were determined to have negative results based on imaging with both tracers (Fig 1). Positive lesions were present in 31 patients with a total of 188 lesions (16 primary tumors, 21 local recurrences, and 151 metastatic lesions). Of these lesions, 26 were confirmed by pathologic diagnosis, and the remaining 162 lesions were evaluated with follow-up imaging (Table 1).

**Table 1:**
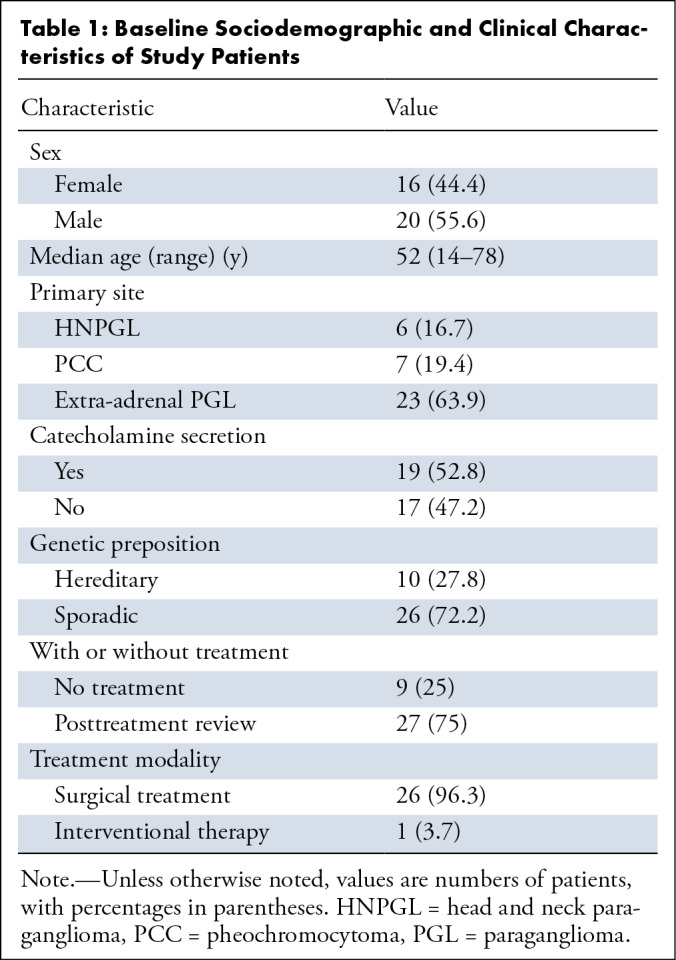
Baseline Sociodemographic and Clinical Characteristics of Study Patients

### Sensitivity of ^68^Ga-DOTANOC and ^18^F-FDOPA PET/CT for Diagnosis of PGLs at Different Sites

There was no evidence of differences in diagnostic sensitivity between the two tracers for PGLs at all anatomic sites. For PCC, the sensitivity values were 96% (27 of 28) for ^18^F-FDOPA and 79% (22 of 28) for ^68^Ga-DOTANOC (*P* = .10). For HNPGL, ^18^F-FDOPA had 85% (17 of 20) sensitivity and ^68^Ga-DOTANOC had 100% (20 of 20) sensitivity (*P* = .23). For extra-adrenal PGL, sensitivities were 65% (91 of 140) and 69% (96 of 140), respectively (*P* = .61) (Table 2).

**Table 2:**
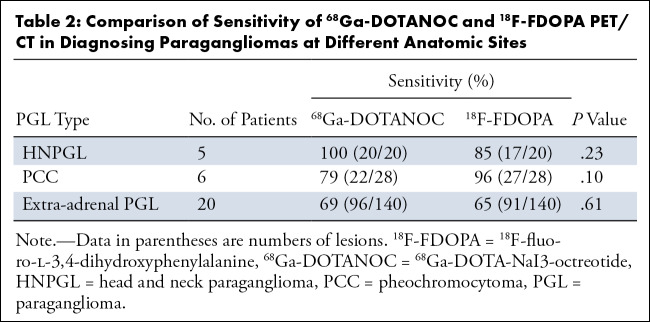
Comparison of Sensitivity of ^68^Ga-DOTANOC and ^18^F-FDOPA PET/CT in Diagnosing Paragangliomas at Different Anatomic Sites

### Diagnostic Performance of ^68^Ga-DOTANOC and ^18^F-FDOPA PET/CT at Lesion Level and Patient Level

**Figure 2:**
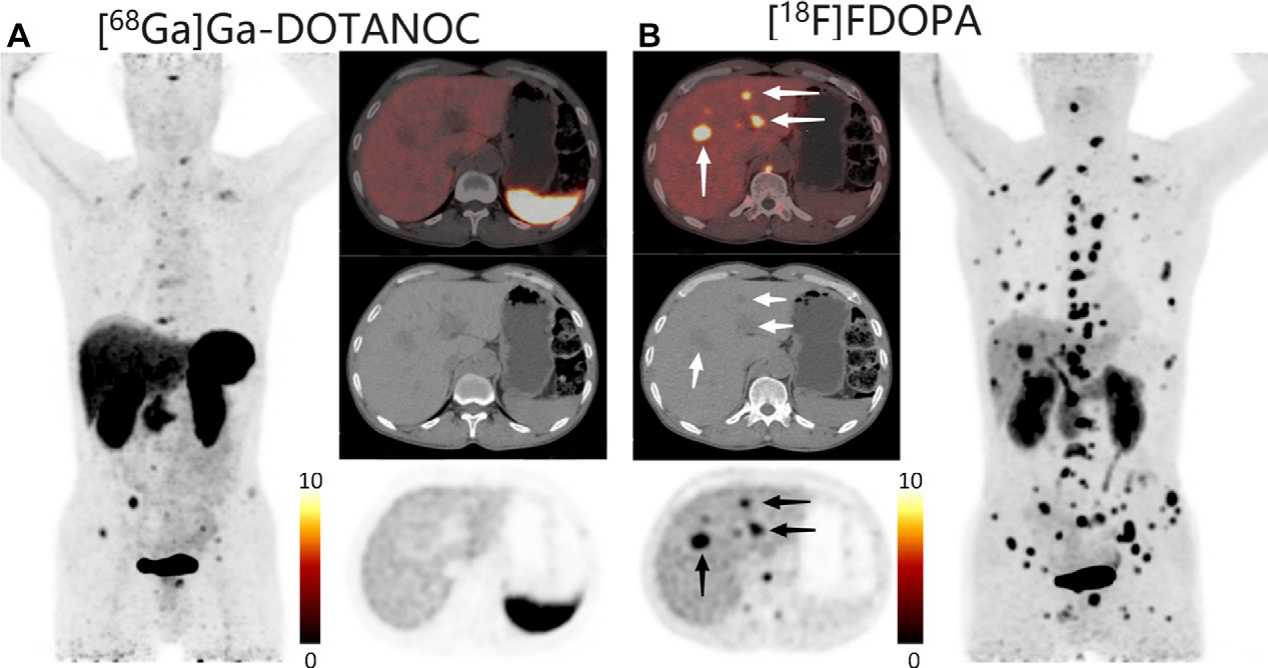
PET/CT scans in a 29-year-old female patient who underwent surgery for retroperitoneal paraganglioma 3 months before reexamination. **(A) **^68^Ga-DOTA-NaI3-octreotide (^68^Ga-DOTANOC) PET/CT scan (axial plane) shows no uptake in the liver. **(B) **^18^F-fluoro-l-3,4-dihydroxyphenylalanine (^18^F-FDOPA) PET/CT scan (axial plane) shows strong uptake in the liver (arrows point to multiple liver metastases).

**Figure 3:**
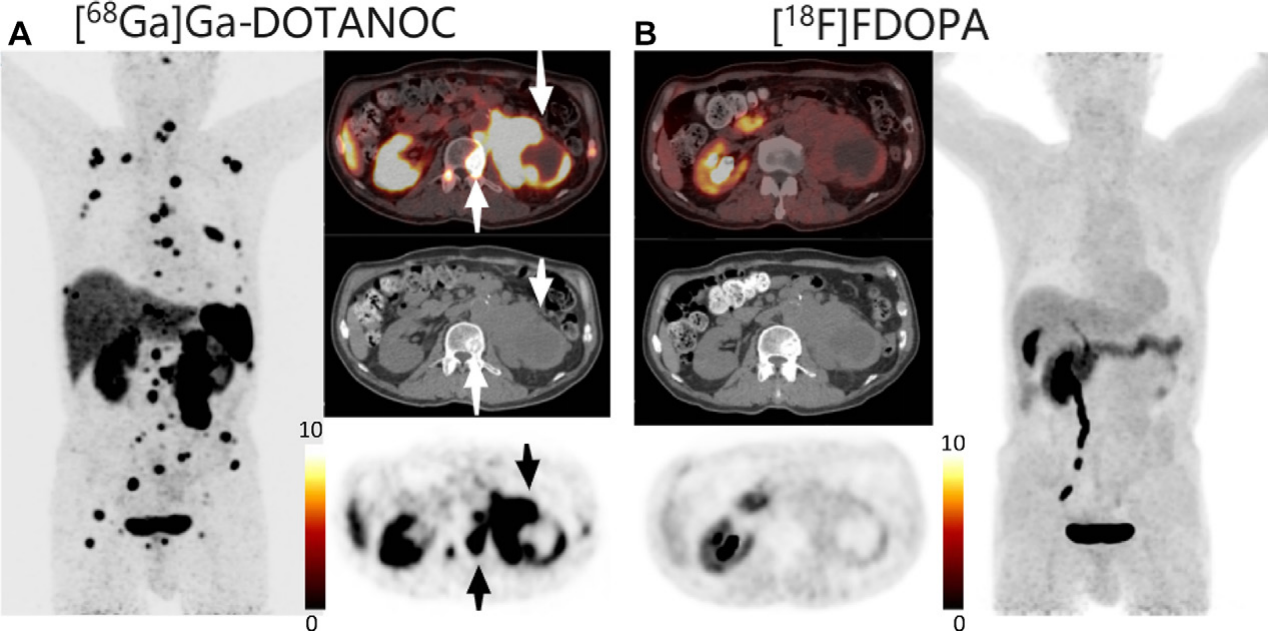
PET/CT scans in a 37-year-old male patient with *SDHx* mutation who underwent retroperitoneal paraganglioma resection 3 years before reexamination. This reexamination showed recurrence of paraganglioma as retroperitoneal and lumbar vertebrae metastases. **(A) **^68^Ga-DOTA-NaI3-octreotide (^68^Ga-DOTANOC) PET/CT scan (axial plane) shows intense uptake in the retroperitoneal and lumbar vertebrae (arrows point to retroperitoneal recurrence and vertebral metastases). **(B) **^18^F-fluoro-l-3,4-dihydroxyphenylalanine (^18^F-FDOPA) PET/CT scan (axial plane) shows no uptake in these same lesions.

In the lesion-level analysis, specificity and accuracy for diagnosing primary tumor or local recurrence were higher with ^68^Ga-DOTANOC than ^18^F-FDOPA PET/CT (specificity, 100% [95% CI: 48, 100] vs 40% [95% CI: 5, 85] [*P* = .03]; accuracy, 91% [95% CI: 77, 97] vs 71% [95% CI: 55, 84] [*P* = .046]). Similarly, for the diagnosis of bone metastases, ^68^Ga-DOTANOC PET/CT had higher sensitivity (97% [95% CI: 90, 100] vs 78% [95% CI: 66, 87]; *P* < .001), negative predictive value (85% [95% CI: 58, 96] vs 42% [95% CI: 32, 53]; *P* = .02), and accuracy (97% [95% CI: 91, 100] vs 81% [95% CI: 70, 89]; *P* < .001) than ^18^F-FDOPA PET/CT. For diagnosis of liver metastases, ^18^F-FDOPA PET/CT had higher sensitivity (73% [95% CI: 52, 88] vs 15% [95% CI: 4, 35]; *P* < .001), negative predictive value (68% [95% CI: 53, 80] vs 41% [95% CI: 37, 45]; *P* = .04), and accuracy (83% [95% CI: 68, 93] vs 46% [95% CI: 31, 63]; *P* < .001) than ^68^Ga-DOTANOC PET/CT (Table 3, Figs 2 and 3).

**Table 3:**
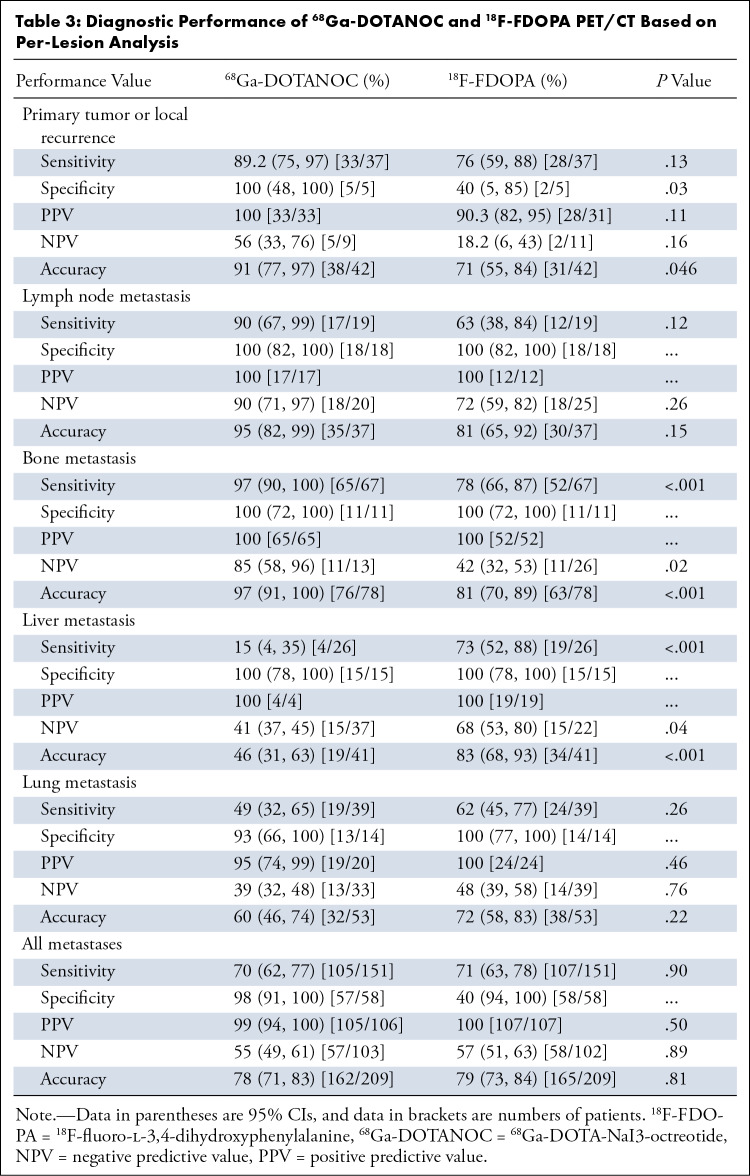
Diagnostic Performance of ^68^Ga-DOTANOC and ^18^F-FDOPA PET/CT Based on Per-Lesion Analysis

In the patient-level analysis, ^68^Ga-DOTANOC PET/CT had higher accuracy for diagnosing primary tumor or local recurrence than ^18^F-FDOPA PET/CT (97% [95% CI: 83, 100] vs 73% [95% CI: 54, 88]; *P* = .03). ^18^F-FDOPA PET/CT had higher sensitivity than ^68^Ga-DOTANOC PET/CT for diagnosing liver metastases (88% [95% CI: 47, 100] vs 25% [95% CI: 3, 65]; *P* = .04) (Table 4).

**Table 4:**
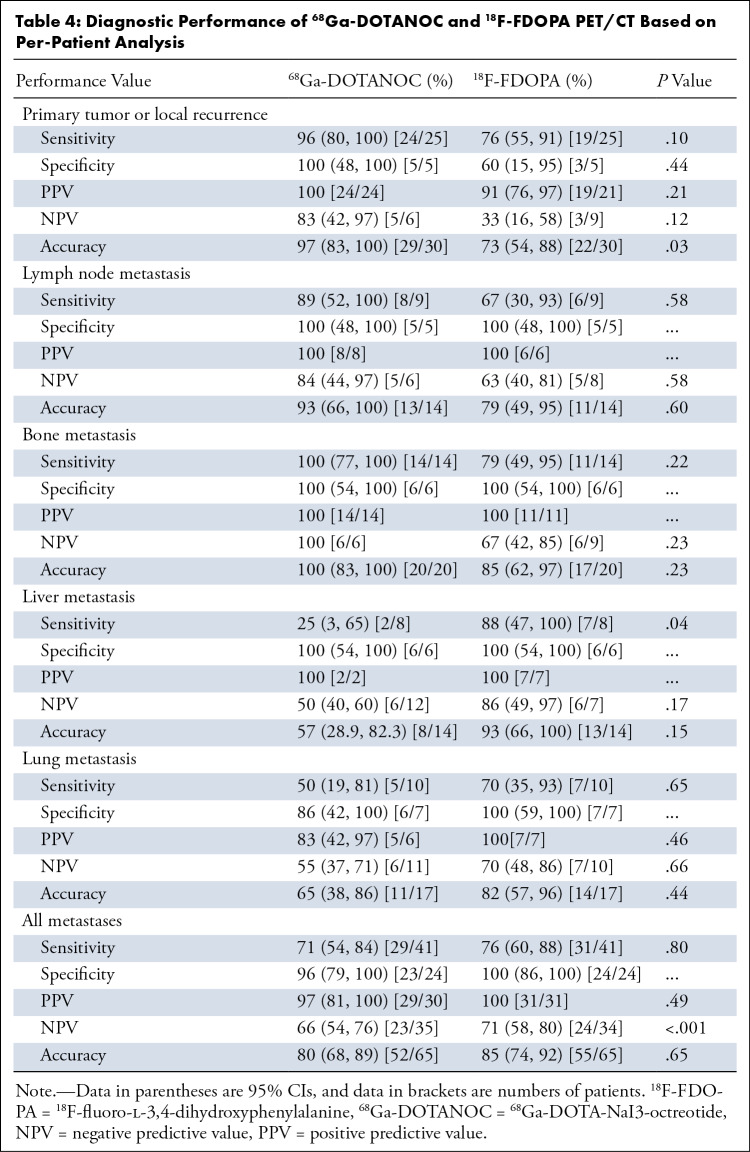
Diagnostic Performance of ^68^Ga-DOTANOC and ^18^F-FDOPA PET/CT Based on Per-Patient Analysis

According to semiquantitative analysis, ^68^Ga-DOTANOC PET/CT had higher SUV_max_ of primary tumor or local recurrence (25.8 ± 22.4 vs 14.0 ± 13.9), bone metastasis (35.6 ± 29.1 vs 18.7 ± 19.9), and liver metastasis (30.8 ± 13.4 vs 11.9 ± 7.2) than did ^18^F-FDOPA PET/CT. The TBR of bone metastasis in ^68^Ga-DOTANOC PET/CT was higher than that in ^18^F-FDOPA PET/CT (42.5 ± 36.0 vs 18.6 ± 20.6; *P* < .001) (Table 5).

**Table 5:**
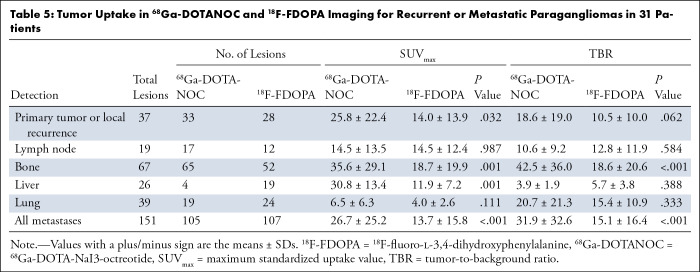
Tumor Uptake in ^68^Ga-DOTANOC and ^18^F-FDOPA Imaging for Recurrent or Metastatic Paragangliomas in 31 Patients

### Sensitivity of ^68^Ga-DOTANOC and ^18^F-FDOPA PET/CT in Patients with *SDHx*-related PPGLs versus Non–*SDHx*-related PPGLs

Ten of the 31 patients with positive lesions had *SDHx* mutations, and 21 had no *SDHx* mutations. In *SDHx*-related PPGLs, there was no evidence of a difference in diagnostic sensitivity between ^68^Ga-DOTANOC PET/CT and ^18^F-FDOPA PET/CT (76% vs 59%; *P* = .18). In non–*SDHx*-related PPGLs, the diagnostic sensitivity of ^68^Ga-DOTANOC PET/CT was lower than that of ^18^F-FDOPA PET/CT (71% vs 84%; *P* = .04).

## Discussion

PPGLs are relatively rare diseases, and few studies have used both ^68^Ga-DOTANOC and ^18^F-FDOPA probes to detect these tumors. To our knowledge, no previous study has evaluated use of ^68^Ga-DOTANOC and ^18^F-FDOPA for postoperative assessment of PGLs. Our study demonstrated that ^68^Ga-DOTANOC PET/CT had higher sensitivity for detecting bone metastases compared with ^18^F-FDOPA PET/CT (97% vs 78%; *P* < .001), whereas ^18^F-FDOPA PET/CT had higher sensitivity for detecting liver metastases than did ^68^Ga-DOTANOC PET/CT (73% vs 15%; *P* < .001).

The HNPGLs in this study (five patients with a total of 20 lesions) were metastatic HNPGLs or postoperative recurrences. Sensitivity of ^68^Ga-DOTANOC PET/CT and ^18^F-FDOPA PET/CT for detecting these tumors did not significantly differ (100% vs 85%, respectively; *P* = .23), which may be due to the small sample size. Although previous studies (12–14) did not directly compare the sensitivity of ^68^Ga-DOTANOC and ^18^F-FDOPA PET/CT for HNPGLs, they found sensitivities of 100% and 97%, respectively. Studies have also shown that ^68^Ga-DOTATOC PET/CT shows similar performance of ^18^F-FDOPA PET/CT in detecting nonmetastatic HNPGLs but better performance in detecting metastatic HNPGLs (sensitivity, 100% vs 56%) (7,9). For diagnosis of PCC in the current study, ^18^F-FDOPA had a high sensitivity of 96.4% but showed no evidence of a difference compared with ^68^Ga-DOTANOC (78.6%; *P* = .10). Previous studies have also shown high sensitivity of ^18^F-FDOPA PET/CT for PCC (95%–100%) (15). This may be due to the lack of uptake by normal adrenal tissue, which gives it an advantage over other radiotracers. Previous research has shown that SUV_max_ significantly differs between involved adrenal glands and normal adrenal glands and that ^18^F-FDOPA PET/CT can help distinguish PCC from normal adrenal glands with the highest diagnostic accuracy (10).

For the diagnosis of liver metastases in our study, ^68^Ga-DOTANOC PET/CT had an exceptionally low sensitivity of 15%. To our knowledge, no previous studies have evaluated performance of ^68^Ga-DOTANOC for detecting liver metastases of PGLs. Under normal conditions, ^68^Ga-DOTANOC is mainly distributed in such organs as the kidney, liver, and spleen and, to a lesser extent, in the brain, heart, and lungs (16). Therefore, although the SUV_max_ of ^68^Ga-DOTANOC lesions is often higher than that of ^18^F-FDOPA lesions, the TBR is actually lower. We believe that relatively high liver SUV_max_ may lead to missed lesions (16,17). Notably, PPGLs are a unique type of tumor that differs from other neuroendocrine tumors of epithelial origin and exhibit substantially higher somatostatin receptor 2 (SSTR2) expression than normal tissues (7). Functionally, SSTR2 can mediate the release of somatostatin, thereby inhibiting endothelial cell proliferation and inducing cell apoptosis. In addition, SSTR2 is involved in antitumor responses in the body, and its low expression may promote tumor cell proliferation and migration (18). Research has shown that highly differentiated tumor cells typically have high SSTR2 expression, whereas poorly differentiated tumor cells have low SSTR2 expression, resulting in lower sensitivity of ^68^Ga-DOTA-SSA probes (19,20). Therefore, ^68^Ga-DOTA-SSA probes for metastatic paragangliomas used in clinical practice may need to be combined with other investigations to exclude the possibility of liver metastases.

The sensitivity of ^18^F-FDOPA PET/CT for bone metastases was lower than that of ^68^Ga-DOTANOC PET/CT. ^18^F-FDOPA biodistribution depends mainly on the distribution and functional status of dopaminergic neurons. FDOPA is predominantly taken up by neurons and used in the dopamine synthesis pathway; PGL bone metastases may not always exhibit this typical FDOPA uptake pattern, resulting in reduced sensitivity (21).

In the present study, the sensitivity of ^18^F-FDOPA PET/CT in patients with *SDHx*-related PPGLs was lower than that in patients with non–*SDHx*-related PPGLs (59% [53 of 90] vs 84% [82 of 98]; *P* < .001). Previous studies (12,22,23) have also demonstrated the higher sensitivity of ^18^F-FDOPA PET/CT in diagnosing non–*SDHx*-related PPGLs versus *SDHx*-related PPGLs, with one study reporting a sensitivity for *SDHx*-related PPGLs of only 45% (24). It has been reported that catecholamine synthesis and storage in tumor cells of PPGLs may be affected because of mutations in *SDHx*, resulting in reduced uptake of ^18^F-FDOPA (15,25,26). Therefore, in clinical practice, one functional imaging examination often cannot depict all lesions present in a patient.

Our study had important limitations. This was a single-center study with a limited number of cases, which may reduce the generalizability of our findings to broader populations. In addition, the potential for selection bias cannot be excluded because all cases were drawn from a single institution, which may have led to a nonrepresentative sample of patients.

In summary, our study comparing the diagnostic performance of ^68^Ga-DOTANOC PET/CT and ^18^F-FDOPA PET/CT in detecting recurrent or metastatic PPGLs showed that ^68^Ga-DOTANOC PET/CT was more sensitive in detecting bone metastases, whereas ^18^F-FDOPA PET/CT was more sensitive in detecting liver metastases. In addition, ^18^F-FDOPA PET/CT may be more suitable for detecting non–*SDHx*-related PPGLs, whereas ^68^Ga-DOTANOC PET/CT showed higher sensitivity in *SDHx*-related PPGLs, although the difference was not statistically significant. In clinical practice, a combined imaging approach using both tracers may provide the most comprehensive detection of PPGL metastases. Moreover, SSTR is a target not only for radionuclide imaging but also for radionuclide therapy for PGLs. Confirmation of receptor affinity by diagnostic imaging can show the potential of peptide receptor radionuclide therapy. Because of the highly heterogeneous nature of PGLs and the abnormalities of catecholamine metabolism in some of the patients, we had hoped to find more lesions that could not be detected with ^68^Ga-DOTANOC PET/CT through ^18^F-FDOPA PET/CT. In the future, we plan to conduct prospective studies with multicenter cooperation to gain a more detailed understanding of the relationship between tumor characteristics and functional imaging.
